# Case report—snare loop retrieval of an unnoticed lost stent after 3 months: the later, the harder

**DOI:** 10.1093/ehjcr/ytaf148

**Published:** 2025-04-08

**Authors:** Farhang Aminfar, David Meier, Stephane Fournier, Vladimir Rubimbura

**Affiliations:** Department of Cardiology, Lausanne University Hospital and University of Lausanne, Bugnon 46, 1005 Lausanne, Switzerland; Department of Cardiology, Lausanne University Hospital and University of Lausanne, Bugnon 46, 1005 Lausanne, Switzerland; Department of Cardiology, Lausanne University Hospital and University of Lausanne, Bugnon 46, 1005 Lausanne, Switzerland; Department of Cardiology, Lausanne University Hospital and University of Lausanne, Bugnon 46, 1005 Lausanne, Switzerland

**Keywords:** Stent loss, Stent retrieval, Snare loops, Complex PCI, Case report, Coronary angioplasty

## Abstract

**Background:**

Stent loss is a rare but serious complication of percutaneous coronary interventions (PCI) that can disrupt coronary flow. This report details the retrieval of an intracoronary stent 3 months after its unnoticed loss during a complex PCI procedure.

**Case Summary:**

A 62-year-old male presented with an inferior STEMI due to right coronary artery (RCA) occlusion, treated by primary PCI. The angiogram revealed a severe calcified stenosis at the left anterior descending artery (LAD)/diagonal bifurcation. In absence of anterior wall motion abnormality, LAD/diagonal stenting was attempted with a DK crush strategy. Despite repeated pre-dilatation, stent delivery to the diagonal branch was unsuccessful. An anchoring balloon attempt led to loss of wire position with guiding catheter extubation and transient chest pain. A septal-left ventricle perforation was evidenced and treated with prolonged balloon inflation. No pericardial effusion was noted. A TIMI 3 flow was achieved in both LAD and diagonal (with an ostial dissection). A follow-up procedure was scheduled three months later. The control angiogram revealed a new intermediate lesion in the left main artery, caused by a dislodged stent extending from the LM to the first diagonal's ostium. Stent retrieval using an EN snare was successful and caused a dissection, necessitating stenting from the LM to proximal LAD.

**Discussion:**

Stent loss during PCI, though rare, requires prompt management through stent crushing or retrieval. Unrecognized stent loss can lead to partial endothelialization, complicating retrieval and causing additional complications. It is thus of utmost importance to always verify stent integrity following unsuccessful delivery to recognize immediately the stent loss.

**Learning points**

- Specific attention should be paid to preparation of calcified coronary lesions prior to stent delivery and the threshold to use intracoronary imaging should be low in case of complex procedures.
- Following failure of stent delivery, its integrity has to be carefully checked systematically.

## Introduction

Over the past decades, the technical improvements as well as increasing operators experience and skills have led to an increasing number of percutaneous coronary interventions (PCI) performed in complex coronary anatomies.^1,2^ Complex and calcified coronary lesions are encountered in about 30% of cases,^3^ and their treatment is associated with a higher risk of complications such as coronary dissection, stent under-expansion, failure of stent implantation and, in some rare instances, stent loss.^4,5^ This latter can be treated with percutaneous retrieval or crushing/jailing behind another stent depending on the configuration, each of which is associated with potential complications such as embolisation, dissection, or perforation.^6–8^ In this report, we describe an initially unnoticed stent loss, with retrieval 3 months after the index procedure in a calcified bifurcation lesion.

## Summary figure

**Figure:**
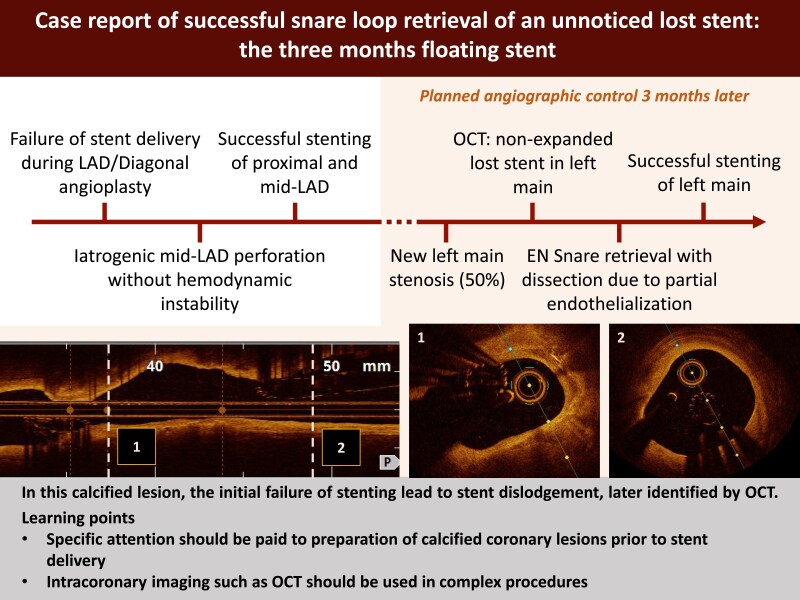


## Case presentation

**Figure 1:**
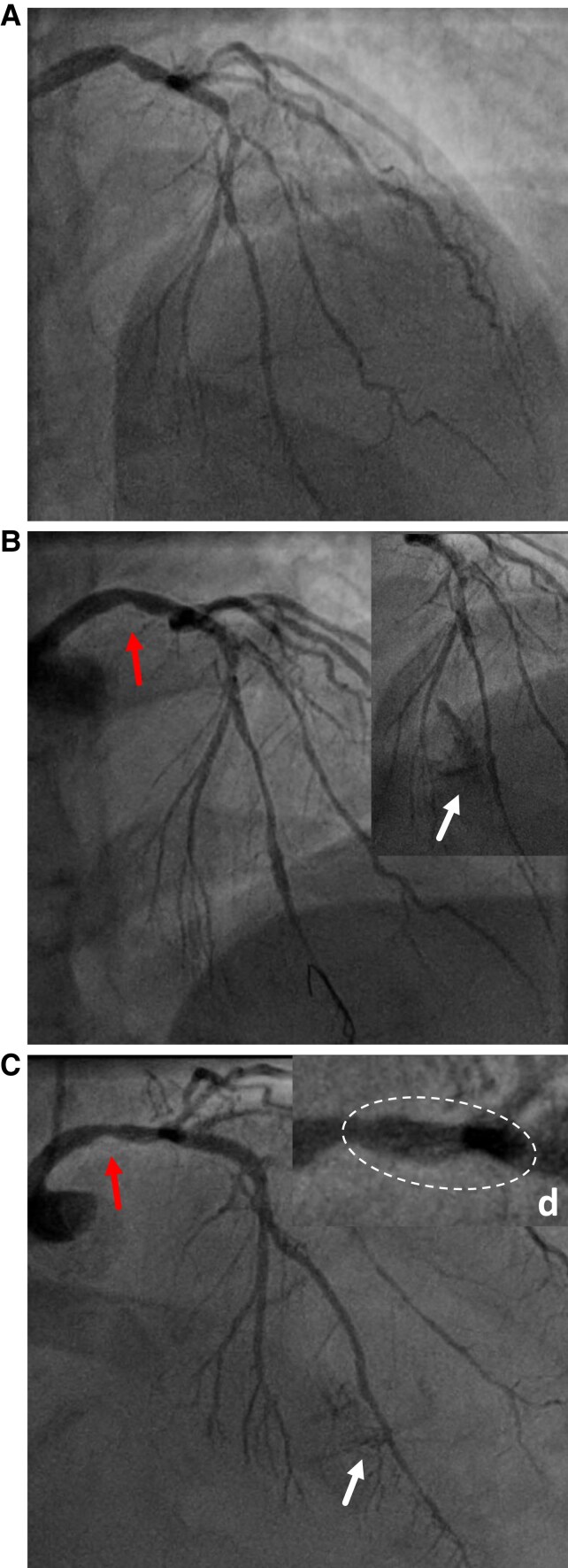
Coronary angiograms evolution. (*A*): initial diagnostic angiogram showing a bifurcation lesion LAD (85%)—first diagonal subocclusion. (*B*): second angiogram was performed on the 10th of September with diagonal stenting failure. The red arrow (left arrow) indicates a non-significant left main stenosis. The white arrow (right arrow) shows the mid-LAD perforation. (*C*): the planned angiogram control at 3 months. The red arrow (left arrow) points at the progression of the left main lesion. The white arrow (right arrow) shows the persistence of mid-LAD—left ventricular communication. (*D*): Zoom on the left main lesion with the non-expanded stent in the white circle.

A 62-year-old male patient, a former smoker with a history of type 2 diabetes, presented to the emergency department with typical chest pain. The ECG showed ST segment elevation in leads II, III, and aVF, and inferior STEMI was diagnosed. On coronary angiogram the culprit lesion was found to be an occluded proximal RCA which was treated with successful implantation of a single drug-eluting stent (DES). Besides, the angiogram identified a very tight complex LAD/diagonal bifurcation lesion (Medina 1-1-1, complex lesion according to DEFINITION criteria^9^ with subocclusive side branch stenosis, moderate to severe calcification and multiple lesions) (*Figure 1A*). Since no anterior wall motion abnormality was observed on echocardiography and in the setting of a very tight diagonal stenosis, a bifurcation angioplasty of the LAD/first diagonal with a DK crush strategy was attempted. Multiple pre-dilatations of the proximal LAD and the first diagonal with semi-compliant and non-compliant balloons of increasing diameters were performed (NC EMERGE, Boston Scientific, USA, 3.0 × 12 mm, at 20 atm in the LAD and NC EMERGE, Boston Scientific, USA, 2.5 × 12 mm, at 20 atm in the diagonal). Despite what appeared to be adequate lesion preparation with multiple balloon dilations and the use of three different stents platforms, implantation of the 2.25 mm stent in the diagonal was impossible due to severe ostial calcification. The use of a guide-extension along with a stiff wire (Grand Slam, ASAHI INTECC, USA) followed by a last attempt with an anchoring balloon (inflated in the mid-LAD) did not allow stent delivery in the diagonal. However, after inflation of an anchoring balloon in the mid-distal-LAD, wire position loss with guiding catheter extubation and transient chest pain occurred. Type III septal-left ventricle perforation was noticed without haemodynamic compromise or pericardial effusion on echocardiographic control (see Supplementary material online, video  *S1*). We believe that the anchoring balloon (NC EMERGE, Boston Scientific, USA, 2.5 × 12 mm) inflated at 12 atm in the mid-LAD led to perforation in the setting of a suspected myocardial bridge at the site of balloon inflation. On angiogram, the perforation appeared to be draining in the left ventricle. This was successfully treated by prolonged inflation of a 2.5 mm balloon in the LAD. Chest pain relief was achieved after LAD stenting and proximal optimisation technique (SYNERGY II, Boston Scientific, USA, 2.5 × 20 mm, at 12 atm). After inflation of the anchoring balloon, we also noticed an ostial diagonal dissection. Since wiring through the dissection plane was challenging, no stent was deployed in the diagonal, and the procedure was ended with TIMI 3 flow in all vessels. Of note, this second part of the procedure was not Optical coherence tomography (OCT)-guided given the significant volume of contrast already used. Because of the difficulties encountered, the patient was scheduled for a repeat angiogram 3 months later with intravascular imaging to exclude any LAD stent deformation or malapposition. He remained asymptomatic in the meantime.

**Figure 2:**
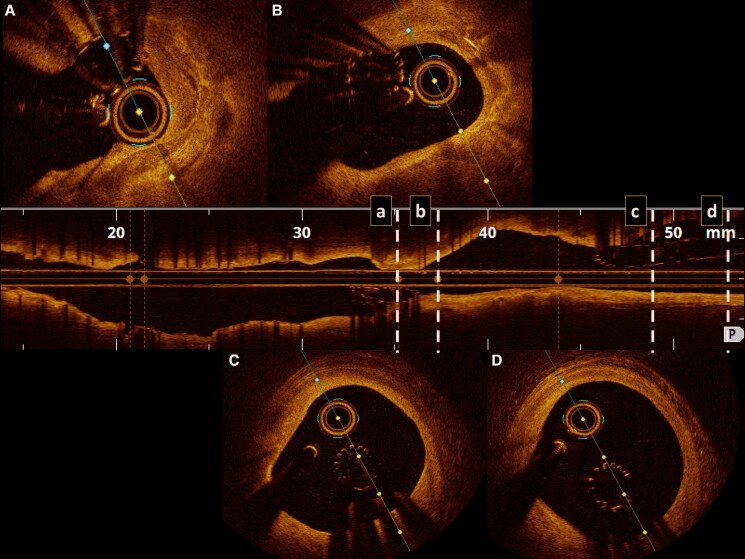
OCT pullback. The lost stent can be identified on the coronal views (*A* to *D*), showing a non-expanded floating stent in the left main (*A*) and (*B*) views, which show the endothelialized portion of the stent.

**Figure 3:**
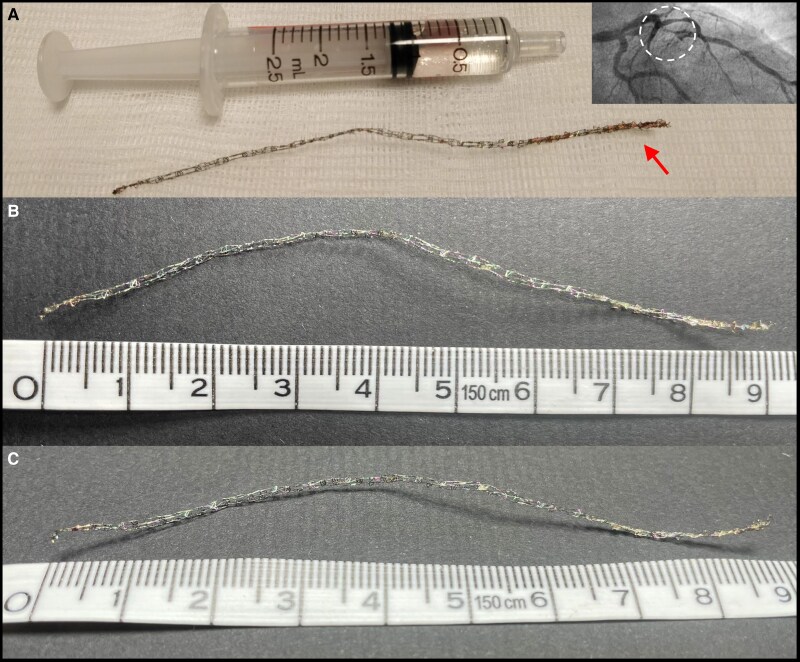
The retrieved stent (*A*) was obtained immediately after retrieval, and the arrow shows the endothelialized portion of the stent that caused iatrogenic dissection once retrieved (dotted circle on the angiogram). The deformation of the stent is shown with (*B*) and (*C*) (measuring scale in centimetres).

The repeat angiogram showed progression of previously identified intermediate left main lesions (*Figure 1B* and *C*, red arrows). Residual septal-to-ventricle communication was also noted (*Figure 1C*, white arrow). An OCT was performed to better assess the left main (LM) lesion and evidenced a lost stent extending from the left main to the first diagonal, that measured at least 35 mm (*Figure 2*). Based on the procedural report from the index PCI, the dislodged stent was identified as an Orsiro mission (Biotronik, Germany) 2.25 × 40 mm. Options were to crush the stent, with the risk of occluding the diagonal that was severely diseased at the ostium, or to try a stent retrieval with a snare. This latter option was chosen, and the use of an EN Snare 3.2F (Merit Medical, USA) allowed a successful retrieval of the stent. However, it caused LAD dissection (*Figure 3A*, dotted circle) extending to the ostium and likely favoured by partial endothelialization of the stent that remained there for 3 months (*Figure 3A*, red arrow). After rewiring of the LAD, a long DES (3.0 × 38 mm) was successfully implanted from the LM to the proximal LAD. The proximal optimisation technique was performed with a good result, both on angiogram and OCT. Post-procedure trans-thoracic echocardiography showed known inferior hypokinesia with preserved ejection fraction, and the patient was discharged after 24 h of monitoring, without any adverse cardiac event. At 12 months, the patient remained asymptomatic with preserved ejection fraction.

## Discussion

The present case describes an unsuccessful stent delivery in a calcified lesion that resulted in unnoticed stent loss. This case illustrates several important aspects of complex PCI that are worth discussing. It highlights the benefit of performing intracoronary imaging in complex bifurcation PCI in accordance with the IA recommendation of the latest guidelines.^10^

Stent loss is a rare complication that is reported in around in 0.5% of stenting procedures^11,12^ and several retrieval techniques have already been reported. However, this situation is generally diagnosed immediately when the stent is not endothelialized and relatively free in the artery. In the present case, the late diagnosis resulted in partial endothelialization of the stent, which was much harder to retrieve, causing a dissection and requiring LM stenting. This highlights the importance of always verifying stent integrity and its presence on the balloon following unsuccessful delivery. Here, the presence of a concomitant LAD perforation probably caused a distraction that led to neglect of this important step. Coronary occlusion and thrombosis following a stent loss have been described but luckily did not occur in this case as the patient was on double antiplatelet therapy, and the scheduled repeat angiogram allowed for ‘early’ recognition of the problem.^6,13^

Additionally, this case reinforces the central role of intracoronary imaging, which could have potentially allowed avoiding some of the issues encountered. First, initial use of OCT or intravascular ultrasound could have identified features of severe calcification warranting the use of advanced calcium modification techniques such as rotational atherectomy.^14^ At the time of the procedure, intravascular lithotripsy or orbital atherectomy were not available in our center. Therefore, lesion preparation was performed mainly with non-compliant balloons. Precise lesion characterisation with intracoronary imaging could have offered better PCI planification, accurate stent diameter selection and optimal lesion preparation before stenting.^15^ Moreover, recent trials, including the OCTOBER trial, have shown improved outcomes with routine use of intravascular imaging for complex PCI.^16,17^ Finally, if OCT had been used following LAD PCI, the lost stent would have been noticed immediately and could have been managed earlier.

## Lead author biography


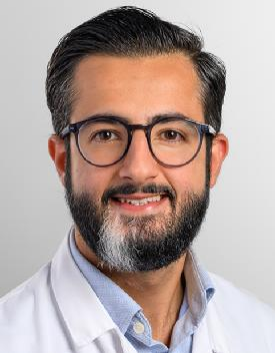


Dr Farhang Aminfar is a resident in the Cardiology department of the University Hospital of Lausanne (Switzerland) with a strong interest in interventional cardiology. He’s involved in coronary physiology with a particular interest in complex coronary lesions.

## Supplementary Material

ytaf148_Supplementary_Data

## Data Availability

The data supporting this article are available within the article and the Supplementary material.

## Notes

**Consent:** The patient has given written informed consent to the present publication, in accordance with COPE guidelines.

**Funding:** None declared.
